# Impact on routine psychiatric diagnostic practice from implementing the DSM-5 cultural formulation interview: a pragmatic RCT in Sweden

**DOI:** 10.1186/s12888-022-03791-9

**Published:** 2022-02-25

**Authors:** Malin Idar Wallin, Maria Rosaria Galanti, Lauri Nevonen, Roberto Lewis-Fernández, Sofie Bäärnhielm

**Affiliations:** 1 Centre for Psychiatry Research, Department of Clinical Neuroscience, Karolinska Institutet, & Stockholm Health Care Services, Stockholm, Region Stockholm Sweden; 2 Department of Global Public Health, Karolinska Institutet, & Centre for Epidemiology and Community Medicine (CES), Stockholm, Region Stockholm Sweden; 3 Department of Medical Sciences, Faculty of Medicine and Health, Örebro University, & Aleris Psychiatry AB, Stockholm, Sweden; 4 Columbia University and New York State Psychiatric Institute, New York, NY USA; 5 Centre for Psychiatry Research, Department of Clinical Neuroscience, Karolinska Institutet & Stockholm Health Care Services, Stockholm, Region Stockholm Sweden

**Keywords:** Cultural formulation, Cultural psychiatry, Clinical assessment, Ethnicity and health

## Abstract

**Background:**

Culture and social context affect the expression and interpretation of symptoms of distress, raising challenges for transcultural psychiatric diagnostics. This increases the risk that mental disorders among migrants and ethnic minorities are undetected, diagnosed late or misdiagnosed. We investigated whether adding a culturally sensitive tool, the DSM-5 core Cultural Formulation Interview (CFI), to routine diagnostic procedures impacts the psychiatric diagnostic process.

**Method:**

We compared the outcome of a diagnostic procedure that included the CFI with routine diagnostic procedures used at Swedish psychiatric clinics. New patients (*n* = 256) admitted to a psychiatric outpatient clinic were randomized to a control (*n* = 122) or CFI-enhanced diagnostic procedure (*n* = 134) group. An intention-to-treat analysis was conducted and the prevalence ratio and corresponding 95% confidence intervals (CI) were calculated across arms for depressive and anxiety disorder diagnoses, multiple diagnoses, and delayed diagnosis.

**Results:**

The prevalence ratio (PR) of a depressive disorder diagnosis across arms was 1.21 (95% CI = 0.83-1.75), 33.6% of intervention-arm participants vs. 27.9% of controls. The prevalence ratio was higher among patients whose native language was not Swedish (PR =1.61, 95% CI = 0.91-2.86). The prevalence ratio of receiving multiple diagnoses was higher for the CFI group among non-native speaking patients, and lower to a statistically significant degree among native Swedish speakers (PR = .39, 95% CI = 0.18-0.82).

**Conclusions:**

The results suggest that the implementation of the DSM-5 CFI in routine psychiatric diagnostic practice may facilitate identification of symptoms of certain psychiatric disorders, like depression, among non-native speaking patients in a migration context. The CFI did not result in a reduction of patients with a non-definite diagnosis.

**Trial registration:**

ISRCTN51527289, 30/07/2019. The trial was retrospectively registered.

## Background

Culture and social context affect the expression and interpretation of symptoms of distress [1–4], potentially complicating transcultural psychiatric diagnostics [1]. Standard instruments may not adequately reflect the experience of depression [5] or other disorders across cultures. The impact of culture can be even more challenging when patient and clinician do not share a native language, as is the case with many migrants and refugees [6]. Diagnostic difficulties increase the risk that mental disorders among ethnic minorities, migrants, and refugees go undetected, are not diagnosed within a reasonable time frame, or are misdiagnosed. Additionally, the quality of the diagnostic assessment may be impaired due to lack of differentiation between primary psychiatric disorders and possible comorbid but secondary conditions. Incorrect diagnoses and poor quality of psychiatric assessment can lead to poor treatment adherence, sub-optimal treatment, and even lack of treatment. There is also the opposite risk: clinicians may misinterpret unfamiliar but non-pathological expressions and behaviours as signs of mental disorder, leading to over-treatment.

Migration and refugee status are risk factors for emotional distress and psychiatric disorders [7–10]. Refugees have a higher risk of common mental disorders related to depression, anxiety, stress, obsessive-compulsive spectrum, and phobias [8, 9]. Migration is also a recognized risk factor for psychotic disorders [11, 12]. Refugees in particular have a higher risk of psychotic disorders, including schizophrenia and other non-affective psychotic disorders, compared to non-refugee migrants [9].

Therefore, the increased risk of distress and psychopathology among migrants and refugees, combined with difficulties in communication, require careful psychiatric assessment. Culturally sensitive assessment tools can meet these clinical diagnostic challenges to some extent. A more comprehensive cultural diagnostic evaluation may facilitate understanding of signs of distress and result in greater differentiation among diagnoses and identification of additional comorbid conditions. The DSM-5 Cultural Formulation Interview (CFI) [1] is a standardized protocol that has been developed to guide an individual cultural assessment during the clinical diagnostic process. It is being widely implemented internationally [13–16]. However, to date, no evaluation has been conducted on the impact of the CFI on diagnostic performance in real-life clinical settings.

## Method

### Aim

This study evaluates whether implementing the core CFI during routine diagnostic procedures leads to changes in the resulting psychiatric diagnoses compared to the usual diagnostic process alone.

### Hypotheses

We hypothesized that:

A larger proportion of patients participating in the CFI (“intervention”) would receive an ICD-10 depressive disorder or anxiety disorder diagnosis, compared to patients engaged only in the usual diagnostic routine (UC, Usual Care).

A larger proportion of patients in the CFI arm would receive multiple diagnoses after the initial diagnostic procedure, compared to those in UC.

A smaller proportion of patients in the CFI arm would still be pending a definite diagnosis (i.e., remain in observation status) after the initial diagnostic procedure, compared to those in UC.

### Study design and setting

The study was designed as an unblinded, individually randomized, controlled parallel trial. In the “intervention” group, the core CFI was added to the routine diagnostic procedures and compared to the usual care (UC) assessment among patients referred to the Järva psychiatric clinic. The area served by the three outpatient clinics included in the study covers a suburban population in western Stockholm with a high proportion of migrants (approximately 50% from outside the Nordic countries compared to 22% in the Stockholm region in general). The average socioeconomic status of the population is low, with approximately twice the proportions of low-income and unemployed persons as in the Stockholm region overall [17].

Patients were given oral and written information, translated into 12 languages, underlining that participation was voluntary and could be withdrawn at any time without negative consequences. Approval was obtained from the Regional Ethical Review Board in Stockholm (2015/243-31/2). Trial registration ISRCTN51527289, 30/07/2019. The trial was retrospectively registered. The target number of participants was 150 in each arm. The trial ended when this target number had been approached for consent.

### Sample

**Fig. 1:**
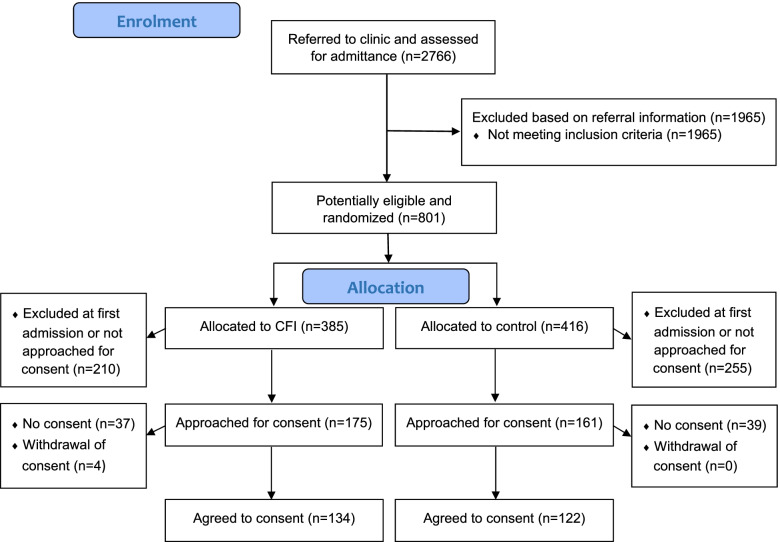
Flow chart of participants’ enrolment

All patients, 18 years of age or older, who had not been in contact with psychiatric services during the preceding 2 years were potentially eligible to participate in the study. Exclusion criteria were: acute suicide risk; inability to give informed consent (e.g. impaired cognition); or age above 65 (to avoid matching clinicians’ clinical competence evaluating older patients, given the small sample of clinicians); and emergency presentations (as randomization was not possible in emergency situations). Referred patients who did not attend the clinical assessment visit were excluded. The inclusion or exclusion was made in two steps. First, the referral was reviewed by a clinical team assigned to assess new referrals, and the exclusion criteria applied at this stage. Second, patients could be excluded during the evaluation if they met clinical exclusion criteria at that point. The same clinical team performed the individual randomization to the intervention or UC arms using a lottery system with equal likelihood of assignment. The patients included in the study were asked for informed consent as early as possible during their clinical consultations. This consent was obtained before the patients were informed about their randomization. Of 801 eligible patients, 385 were randomized to the intervention group and 416 to the UC group (Fig. 1). The final sample of consented patients consisted of 134 intervention and 122 UC participants.

### Diagnostic procedures

Patients in the intervention arm participated in the core CFI, without CFI supplements [1], in addition to the standard psychiatric diagnostic procedure, which was the same for both arms. In psychiatric services in the Stockholm region, the mandatory standardized diagnostic procedure (SDP) [18] for new patients includes social and clinical anamneses, two assessment tools – the Mini-International Neuropsychiatric Interview (MINI) and Clinical Global Impression (CGI) scale – and three web-based self-administered screening tools – the Alcohol Use Disorder Identification Scale (AUDIT-C), Patient Health Questionnaire, 9-item version (PHQ-9), and Adult ADHD Self-Report Scale (ASRS). An ICD-10 diagnosis must be determined and included in the patient’s health record within three clinical encounters. The SDP can be performed by various professionals; however, the final diagnostic evaluation and assignment of ICD-10 codes is performed by a psychiatrist, or occasionally a psychologist, at the end of the SDP, and documented in the health record. The diagnostic categorisation is based on information gathered in the SDP, often complemented by information exchange within the professional team. This procedure was followed in this study for both groups, while for the intervention group the CFI information was also included. The patients’ narrative answers to the CFI questions were documented in detail in the electronic health record.

Two independent groups of clinical staff (*n* = 15 per group) oversaw the diagnostic procedures. Intervention group clinicians consisted of 10 psychologists/psychotherapists, 3 psychiatric nurses, 1 counsellor, and 1 mental health auxiliary. UC group clinicians were 10 psychologists/psychotherapists, 4 psychiatric nurses, and 1 occupational therapist. The clinicians were assigned to the study arms by the outpatient clinic managers to maintain a fair balance of professions and clinical experience. In both groups, 85% of the SDPs were performed by a psychologist/psychotherapist. The clinicians performing the SDP in the intervention arm received special training on the CFI, including lectures, role-plays using the CFI, and discussions of clinical cases. In both arms, the clinical staff had extensive experience working with multi-cultural populations.

### Outcome

The primary outcome was the prevalence of selected diagnoses, described below, at the conclusion of the standardized diagnostic procedure. We compared the prevalence of diagnoses of depressive (ICD-10 F32-F33) and anxiety disorders (ICD-10 F40-F41) identified after the SDP, as they are the most common diagnoses among newly referred patients. Other disorders, such as PTSD, were not analysed because of their small proportion in this sample. As secondary outcomes, we compared the proportions of patients who were still under observation, without an identified diagnosis, at the end of the SDP (delayed diagnosis) and the prevalence of multiple comorbid diagnoses. The effect of the intervention was defined as the prevalence ratio of the above outcome measures between study conditions. Patients whose native language was not Swedish were identified for separate analyses.

### Data collection and analysis

Data were extracted from electronic health records for visits between August 2015 and May 2017 by the first author (MIW), who was not blinded to participants’ group assignment. Diagnostic data were collected as formal ICD-10 codes documented by the clinicians in the patient’s health record after the mandatory SDP was completed. To control for information bias, diagnostic ICD-10 codes were confirmed by the last author (SB).

### Statistical analyses

The primary statistical analysis was conducted as intention-to-treat, including all eligible patients randomized to the intervention (*n* = 134) or UC (*n* = 122) arms. As a measure of association, we calculated the prevalence ratio (PR) and corresponding 95% confidence intervals (CI) of diagnoses of depressive and anxiety disorders, of multiple diagnoses, and of delayed diagnoses at the end of the respective diagnostic procedures. Then, the same analyses were conducted by stratifying patients into two language groups, native Swedish speakers and non-Swedish native speakers, to test whether the CFI might be more useful in clinical situations potentially affected by language difficulties. Some patients were referred to the clinic with a notation indicating the referring clinician’s suspicion of a specific diagnosis. To control for possible preconceptions about primary diagnoses of depression or anxiety disorder from referral information, subgroup analyses were conducted for patients with and without suspicion of diagnosis upon referral, including patients’ native language status. Finally, to ensure equivalent assessment quality between the two arms, separate analyses were conducted for two SDP components (MINI and PHQ-9) and for appointments with a psychiatrist vs. other professionals, using prevalence ratio or mean/median estimates. All analyses were conducted with SAS version 9.4 (SAS institute inc, see https://www.sas.com). Statistical significance was set at *p* = 0.05 throughout.

## Results

Over half of the participants grew up in Sweden or another Nordic country (61.7%) or were native Swedish speakers (53.9%) (Table 1). An interpreter assisted during the consultations of 14 (10.4%) intervention participants and 8 (6.5%) UC patients. There were no major differences across groups with respect to sex, country of origin, number of years in Sweden (among migrants) and native language (Table 1). Intervention participants had a higher level of education and were slightly older than the UC group. UC participants were more likely to be unemployed, but intervention group participants were more likely to receive welfare benefits. Information about the referring agency was missing more frequently in the intervention group than in the UC group; however, after missing values were excluded there was no difference between the two groups in this regard.

**Table 1:** Baseline characteristics of the sample, by study arm (*N* = 256)

	Intervention (CFI) N134 (%)	Usual care (UC) N122(%)	Total N256(%)
Sex:			
Women	86(64.2)	73(59.8)	159(62.1)
Men	47(35.1)	49(40.2)	96(37.5)
Missing	1(.7)	.	1(.4)
Age group:			
17-24	37(27.6)	32(26.2)	69(27.0)
25-34	34(25.4)	40(32.8)	74(28.9)
35-44	24(17.9)	25(20.5)	49(19.1)
45-64	38(28.4)	24(19.7)	62(24.2)
Missing	1(.7)	1(.8)	2(.7)
Employment group:			
Employed	58(43.3)	55(45.1)	113(44.1)
Not employed	31(23.1)	36(29.5)	67(26.2)
Welfare benefits	40(29.9)	28(23.0)	68(26.6)
Missing	5(3.7)	3(2.5)	8(3.1)
Educational level:			
Elementary school or lower	25(18.7)	39(32.0)	64(25.0)
College	28(20.9)	22(18.0)	50(19.5)
Senior high school	53(39.6)	41(33.6)	94(36.7)
Missing	28(20.9)	20(16.4)	48(18.8)
Referral diagnosis:			
Suspicion of psychiatric diagnoses	91(67.9)	94(77.0)	185(72.3)
Generic problem description	35(26.1)	27(22.1)	62(24.2)
No indication	8(6.0)	1(.8)	9(3.5)
Referring agency:			
Voluntary admission	29(21.6)	36(29.5)	65(25.4)
Referral from primary care clinics	69(51.5)	52(42.6)	121(47.3)
Referral from other caregivers	28(20.9)	34(27.9)	62(24.2)
Missing	8(6.0)	.	8(3.1)
Country of origin^a^			
Middle east	15(11.2)	14(11.5)	29(11.3)
Other European countries	16(11.9)	9(7.4)	25(9.8)
Sweden or other Nordic countries	85(63.4)	73(59.8)	158(61.7)
Other	12(9.0)	15(12.3)	27(10.5)
Missing	6(4.5)	11(9.0)	17(6.6)
Mother tongue:			
Swedish	70(52.2)	68(55.7)	138(53.9)
Non-Swedish	57(42.5)	46(37.7)	103(40.2)
Missing	7(5.2)	8(6.6)	15(5.9)
Number of years in Sweden (when country of origin not Sweden):	M (SD) 17.8 (11.1)	M (SD) 16.9 (10.3)	17.35(10.6)

At the completion of the diagnostic assessment, 33.6% of participants in the intervention arm, vs. 27.9% of UC patients, received a diagnosis of depressive disorder (Table 2), resulting in a prevalence ratio (PR) of 1.20 (95% CI = 0.83-1.75). This was higher when the analysis was restricted to patients whose native language was not Swedish (PR =1.61, 95% CI = 0.91-2.86).

**Table 2:** Diagnoses after completion of the diagnostic procedure (intention-to-treat analysis)

	Intervention		Usual care (UC)		Total		Prevalence ratio and 95% CI
Diagnosis	N	N(%)	N	N (%)	N	N (%)	CFI/UR
Depressive disorders (ICD-10 F32-F33)							
All patients	134	45(33.6)	122	34(27.9)	256	79(30.9)	1.20 [0.83-1.75]
Native language other than Swedish	57	24(42.1)	46	12 (26.1)	103	36 (35.0)	1.61 [0.91-2.86]
Native Swedish speakers	70	20(28)	68	22(32.4)	138	42(30.4)	0.88 [0.53-1.46]
Anxiety disorders (ICD-10 F40-F41)							
All patients	134	38(28.4)	122	35(28.7)	256	73(28.5)	0.99 [0.67-1.46]
Native language other than Swedish	57	13(22.8)	46	13(28.3)	103	26(25.2)	0.81 [0.42-1.57]
Native Swedish speakers	70	23(32.9)	68	19(27.9)	138	42(30.4)	1.18 [0.71-1.95]
Multiple diagnoses							
All patients	134	27(20.1)	122	29(23.8)	256	56(21.9)	0.85 [0.53-1.35]
Native language other than Swedish	57	19(33.3)	46	9(19.6)	103	28(27.2)	1.70 [0.85-3.40]
Native Swedish speakers	70	8(11.4)	68	20(29.4)	138	28(20.3)	0.39 [0.18-0.82]
Delayed diagnosis (continued observation)							
All patients	134	36(26.9)	122	29(23.8)	256	65(25.4)	1.13 [0.74-1.73]
Native language other than Swedish	57	18(31.6)	46	11(23.9)	103	29(28.2)	1.32 [0.70-2.51]
Native Swedish speakers	70	14(20.0)	68	13(19.1)	138	27(19.6)	1.05 [0.53-2.06]

Across all patients (Table 2), the prevalence of anxiety disorder diagnoses was nearly identical in the intervention (28.4%) and UC groups (28.7%) (PR = .99, 95% CI = 0.67-1.46). The stratification by native language yielded higher prevalence of anxiety disorders in the intervention group among native Swedish speakers, contrary to our findings for depressive disorder.

The prevalence ratio of multiple diagnoses was similar in the intervention (23.8%) and the UC groups (21.9) (PR = .85, 95% CI = 0.53-1.35). The number of multiple diagnoses was higher for the intervention group in the subset of patients who were non-Swedish native speakers, while the reverse was true to a statistically significant degree among native Swedish speakers (PR = .39, 95% CI = 0.18-0.82) (Table 2).

Using the CFI in the initial assessment did not result in a reduction in the proportion of patients without a definite diagnosis at the conclusion of the initial diagnostic procedure (PR = 1.13, CI = 0.74-1.73). The proportion of patients without a definite diagnosis was higher in the CFI group among non-native Swedish speaking patients (PR = 1.32, CI = 0.70-2.51) compared to native Swedish speakers (PR = 1.05, CI = 0.53-2.06) (Table 2).

We assessed the prevalence of diagnostic outcomes among participants who were referred without suspicion of a specific diagnosis (*n* = 43 CFI and *n* = 28 UC; not included in tables). Assignments of depressive, anxiety, multiple, and delayed diagnoses were similar for this group across the two study arms overall, but the proportion of individuals with a particular diagnostic outcome in the group without a specific diagnostic suspicion at baseline was higher among non-Swedish native speakers in the CFI arm for each diagnostic condition: 36.8% vs. 30.0% (depression), 31.6% vs. 20.0% (anxiety), 36.8% vs. 10.0% (multiple), and 36.8% vs. 20.0% (delayed), respectively.

To confirm the equivalence of diagnostic procedures apart from the CFI across study conditions, we compared the prevalence during the initial mental health assessment of being evaluated by a psychiatrist (vs. only another professional), of using the MINI, and of PHQ-9 scores. There were no between-group differences in the percentage of appointments with a psychiatrist, the frequency of using the MINI, or in PHQ-9 scores calculated as a median (16 and 16, respectively) or a mean (14.6 and 15.2, respectively) (Table 3).

**Table 3:** Key assessment instruments and procedures used in the diagnostic assessment

Assessment instruments and procedures	Intervention		Usual care (UC)		Total		Prevalence ratio and 95% CI
	N	Instrument/procedure N(%)	N	Instrument/procedure N(%)	N	Instrument/procedure N(%)	
The assessment included an appointment with a psychiatrist	134	105(78.4)	122	98(80.3)	256	203(79.3)	0.98 [0.86-1.11]
MINI conducted^a^	134	44(32.8)	122	36(29.5)	256	80(31.3)	1.11 [0.77-1.60]
PHQ-9 score	**M (SD)** 14.6(6.8)	**Med(Var)** 16(45.8)	**M (SD)** 15.2(6.6)	**Med(Var)** 16(43.0)	**M (SD)** 14.9(6.7)	**Med(Var)** 16(44.3)	

^a^The Mini International Neuropsychiatric Interview

## Discussion

### Main findings

In this pragmatic randomized controlled trial, we compared the usual assessment process with a novel diagnostic procedure that included a culturally sensitive interview protocol, in three psychiatric outpatient clinics serving a multicultural catchment area in Stockholm, Sweden.

The CFI had a small effect on diagnostic categorization but varied by diagnostic group. Compared to the usual care (UC) group, patients who were assessed using the CFI were slightly more likely to receive a diagnosis of depressive disorder. This effect was due to the impact of the CFI on the assessment of patients whose native language was not Swedish. The associations were not statistically significant, possibly due to the small sample size. By contrast, no effect from the CFI on the detection of anxiety disorders was observed in the overall sample.

Contrary to our hypothesis, we did not find a higher prevalence of multiple comorbid diagnoses when using the CFI in the overall sample. Instead, we found that native language modified the association in the same way as for depressive disorder diagnoses: higher proportion of diagnosed patients in the group whose native language was not Swedish and lower proportion among native Swedish speakers; this finding attained statistical significance in the latter group. Among patients whose native language was not Swedish it appears that the CFI contributed to improved identification of comorbidity. Also contrary to our hypothesis, use of the CFI did not result in a reduction in the proportion of patients without a definite diagnosis at the conclusion of the initial diagnostic procedure. The proportion of patients without a definite diagnosis was higher among non-native Swedish speakers, while there was no association among native Swedish speakers.

Our findings suggest that using the CFI may affect the diagnostic categorization in diverse ways among different groups of patients. This correlates with findings from an earlier study in which a culturally sensitive assessment showed diverse effects on diagnostic categorization among different subgroups of patients in a study population with psychotic disorder [19]. The language-associated findings in our study are similar to those in an intervention trial of shared decision-making, which showed a stronger effect in linguistically discordant patient-clinician relationships [20].

There appears to be a small advantage of the CFI in detecting depression and multiple diagnoses among the non-Swedish speaking patients, indicating that it could be particularly helpful in diagnostic evaluation of symptoms in migrant groups. Previous studies have raised serious questions about the conceptual appropriateness of applying diagnostic systems to migrants whose language of distress does not incorporate conventional psychiatric symptoms commonly found within diagnostic systems [2, 21]. Symptoms of mental health problems vary across ethnic groups, possibly contributing to a potential underestimation of their occurrence in certain population subgroups. Varying symptoms may be associated with alternative explanatory models of illness that may also affect assessment of the prevalence of mental disorders [22]. Local and culturally relevant instruments can capture salient features of disorders (e.g. depression), not identified through ordinary clinical instruments [23].

The improved identification of depression and comorbidity in the non-native-speaking group of patients could be due to the culturally sensitive information contributing to a more thorough assessment. Also, the questions might help the patients to describe their situation in greater detail, using their own clinical terms when describing their problems and resulting in a more nuanced clinical characterization. Our higher prevalence of depression diagnoses may represent an undetected overrepresentation of depression disorders at referral among non-native Swedish speakers that was identified in clinical situations where the CFI was used.

In a study performed after this RCT was completed, the clinicians in the intervention arm were interviewed about their experience of using the CFI. They said that they approached the patients’ problems in a new manner and that this affected their clinical reasoning and assessment [24]. It is possible that the narrative approach of the CFI facilitated clinicians’ identification of some psychiatric symptoms, such as depression, among non-native Swedish speaking patients. The CFI did not add a diagnostic advantage among the native Swedish speakers, probably because their more classical symptom presentation was more familiar or obvious to the clinicians. Why we did not find a higher prevalence of anxiety disorders among non-native Swedish speaking patients remains to be explained.

Our expectation, that integrating the CFI in the ordinary diagnostic process would lead to a more rapid completion of the diagnostic assessment, was not supported; rather, our results point to the opposite. This could be because the answers provided by the CFI required a longer time for analysis, leading to the need for supplementary information. This may have prolonged the assessment process and postponed the diagnostic categorization. This explanation is speculative but deserves to be explored in future studies. According to the SDP procedure in psychiatric care in Stockholm, a diagnostic categorization should be made within 3 clinical appointments, in this study expanded to 4. We do not know what the potential proportions of delayed diagnosis would be with an extended timeframe.

Our findings are not explained by different proportions of patients referred with a suspicion of depressive or anxiety disorder, by differences in use of routine diagnostic instruments (MINI, PHQ-9), or in the proportion of assessments conducted by a psychiatrist. However, it is possible that our findings were affected by the CFI having a greater impact among patients who were referred without the suspicion of a specific diagnosis. The CFI may have contributed information that helped identify symptoms of depression and comorbidity among non-native Swedish-speaking patients in particular. This would reproduce the results of an earlier study in which a culturally sensitive assessment tool helped refine diagnostic assessments and enhanced the identification of comorbidity [25].

Our findings of the CFI impact on diagnostic categorization are small and could be due to chance. Almost none of the associations were statistically significant, revealing the difficulty of attempting to detect small effects with a relatively small sample. Further, clinicians’ extensive experience with multi-cultural populations at our study sites may also have reduced an added advantage of using the CFI.

The CFI effects on two of the four diagnostic outcomes were in the direction we hypothesized, based on our understanding of the expected higher impact of CFI in the group whose native language is not Swedish. The CFI was associated with the other two diagnostic outcomes in opposite patterns.

Our findings may also be due to the generic effect of adding another diagnostic instrument to the assessment, regardless of the specific cultural focus of the CFI [19]. Since the staff involved in the diagnostic procedure was not blinded to patient assignment, we cannot exclude the possibility that patients receiving the CFI underwent a more careful assessment of their symptoms, leading to a more accurate and complete psychiatric diagnosis. However, an attention bias should translate into an enhanced diagnostic process across disorders in the CFI arm, including an equally decreased proportion of patients with diagnostic delay, which was not the case in this study. The risk of information bias is also a possibility because researchers might be more motivated to find diagnostic information in the intervention patients’ health records leading to higher extraction of ICD codes in the CFI arm. However, we controlled for this risk by having the last author (SB) review the chart abstractions.

In this clinical context, the CFI was never performed by the clinicians making the final diagnosis, but by other members of the clinical team, elevating the importance of team communication and accuracy when collecting information from the health record. Although responses to each CFI question were documented in the health record, it is unclear how the psychiatrists weighed this information when formulating the diagnosis. Differential attention across psychiatrists to the CFI documentation could contribute to the variation observed in the effect of the CFI and may, together with a wealth of experience among the clinicians in multicultural assessments, explain why the CFI did not have a larger effect on diagnostic categorization. Engaging the psychiatrists in how to utilise the CFI documentation and formulate a couple of questions in their own practice about the patient’s understanding of illness, based on the CFI data, might result in an active engagement with the CFI information.

### Strengths and limitations

This study, to the best of our knowledge, is the first to evaluate the effect of the CFI on psychiatric diagnostic categorization. The study took place in a naturalistic setting, with advantages and disadvantages. One advantage is that the core CFI was evaluated in a real-life context with associated challenges, such as heavy workload for clinicians, high influx of new patients, clinician turnover, and restricted time for communication between professionals. Many patients were excluded at first admission or never approached for consent, which may be a limitation of the study, although the numbers of excluded patients were comparable between groups. The number of patients who did not give consent, or withdrew their consent, was small. Also, an interpreter was used in almost twice as many consultations in the intervention group as in the UC group, although these numbers were small. How this might have affected the results is uncertain.

An important limitation was that CFI was not conducted by the psychiatrists in charge of the formal diagnostic categorization, and we lack information on how they weighed the CFI information in their deliberations. This diagnostic process corresponds with clinical praxis in Swedish psychiatric outpatient settings. The division of tasks in clinical teams is common, constituting an area for further development in the implementation of the CFI and a potential limitation of its diagnostic advantage. An additional limitation is that the researcher extracting the diagnostic evaluations from clinical files were not blinded to patients’ study assignment.

A strength of the study in terms of internal validity is that the psychiatrists who made the final formal diagnostic categorization did not receive any training in the CFI, preventing inequalities in training from affecting the diagnostic results. However, this may have reduced the effect of the CFI. The clinicians in both groups were experienced in working in a multicultural area, which possibly increased the acceptability of the CFI.

## Conclusion

The results suggest that the implementation of the DSM-5 CFI in routine psychiatric diagnostic practice may facilitate identification of symptoms of certain psychiatric disorders, like depression, among non-native speaking patients in a migration context. However, the CFI did not result in a reduction of patients with a non-definite diagnosis at initial evaluation. When the CFI is not conducted by the same staff assigning the final diagnosis, the diagnostic advantage of the CFI, when added to the usual procedure, may be limited without further work on how to integrate it in the diagnostician’s practice. Further evaluations of the implementation of the CFI in DSM-5 in psychiatric clinical care and praxis are needed.

## Data Availability

The datasets generated and analysed during the current study are not publicly available due to confidentiality of medical records, but statistical analyses are available from the corresponding author on reasonable request.

## Abbreviations

CFI: Cultural Formulation Interview
UC: Usual care
SDP: Standardized diagnostic procedure
MINI: the Mini-International Neuropsychiatric Interview
CGI: Clinical Global Impression
AUDIT-C: The Alcohol Use Disorder Identification Scale
PHQ-9: Patient Health Questionnaire, 9-item version
ASRS: Adult ADHD Self-Report Scale
